# Soft Coral *Dendronephthya puetteri* Extract Ameliorates Inflammations by Suppressing Inflammatory Mediators and Oxidative Stress in LPS-Stimulated Zebrafish

**DOI:** 10.3390/ijms19092695

**Published:** 2018-09-10

**Authors:** Eun-A Kim, Yuling Ding, Hye-Won Yang, Soo-Jin Heo, Seung-Hong Lee

**Affiliations:** 1 Jeju International Marine Science Center for Research & Education, Korea Institute of Ocean Science & Technology (KIOST), Jeju 63349, Korea; euna0718@kiost.ac.kr (E.-A.K.); sjheo@kiost.ac.kr (S.-J.H.); 2 Department of Pharmaceutical Engineering, Soonchunhyang University, Asan 31538, Korea; dingyuling@naver.com; 3 Department of Marine Life Sciences, Jeju National University, Jeju 63243, Korea; koty221@naver.com

**Keywords:** soft coral, *Dendronephthya puetteri*, anti-inflammatory effect, zebrafish model

## Abstract

Marine-derived extract and/or bioactive compounds have attracted increasing demand due to their unique and potential uses as cures for various inflammation-based diseases. Several studies revealed anti-inflammatory candidates found in soft corals. However, the effects of soft corals on inflammation in an in vivo model remain to be determined. Therefore, the extract of soft coral *Dendronephthya puetteri* (DPE) was investigated for an in vivo anti-inflammatory effect in a lipopolysaccharide (LPS)-stimulated zebrafish model to determine its potential use as a natural anti-inflammatory agent. We also investigated whether DPE has toxic effects in a zebrafish model. No significant changes were observed in terms of survival, heart beat rate, or developmental abnormalities in the zebrafish embryos exposed to a concentration below 100 µg/mL of DPE. Treating the zebrafish model with LPS-treatment significantly increased the ROS, NO generation, and cell death. However, DPE inhibited this LPS-stimulated ROS, NO generation, and cell death in a dose-dependent manner. In addition, DPE significantly reduced the mRNA expression of both iNOS and COX-2 and markedly suppressed the expression levels of the proinflammatory cytokines, TNF-α and IL-6, in an LPS-stimulated zebrafish model. These findings demonstrate that DPE has profound anti-inflammatory effect in vivo, suggesting that DPE might be a strong natural anti-inflammatory agent.

## 1. Introduction

Inflammation is a complex stereotypical response of the body to cell damage and tissue vascularization. However, excessive and uncontrolled inflammation is detrimental to all tissues, as it may cause a variety of inflammatory disorders and human diseases [1,2,3]. Therefore, inhibition of excessive inflammation is important for reducing the risk of inflammation-derived diseases. In addition, recently, skin therapeutics highlight combinational treatment, like the use of moisturizers, antibiotics, and anti-inflammatory agents, for the treatment of skin inflammation [4]. Hence, for the development of anti-inflammatory therapeutics, interest in the discovery of natural products has risen exponentially, and there has also been increasing awareness of the benefits of natural anti-inflammatory properties.

Many marine organisms have unique structures that are not found in terrestrial organisms. Of these marine organisms, marine invertebrates have emerged as a focus for research due to their diverse chemical structures and interesting biological activities. Soft corals are a group of colonial invertebrates that form a set of marine organisms that occur widely in the coral reefs throughout the world [5,6]. Among the Alcyonacean soft corals, the genus, *Dendronephthya*, is one of the most widely distributed soft corals genera throughout the tropical coastal water of the Indian Ocean, Pacific Ocean, and that around Southeast Asia [7]. *Dendronephthya* species have been recognized as rich sources of novel and diverse chemical structures with a broad spectrum of bioactive functionalities [8,9,10]. Recently, the number of Alcyonacean soft coral populations in the sea of Jeju Island, Korea’s southernmost island, has been increasing due to tropical weather. Several studies have recently demonstrated that *Dendronephthya* species collected from Jeju Island display a range of biological activities [11,12,13]. Although such results indicate the potential of the soft coral *Dendronephthya* species as natural bioactive candidates, *Dendronephthya puetteri* collected from the sea of Jeju Island has not been extensively studied in terms of toxicity and anti-inflammatory activities in an in vivo model.

The vertebrate zebrafish (*Danio rerio*) is a small tropical freshwater fish, which has emerged as a useful vertebrate model organism because of its small size, large clutches, transparency, low cost maintenance, and morphological and physiological similarity to mammals [14,15]. Owing to these advantages, the value of the zebrafish as a model organism for in vivo drug toxicity and efficacy studies has recently come to be recognized [16,17,18]. In addition, the optical transparency of zebrafish embryos allows for noninvasive and dynamic imaging of the inflammation in vivo. Therefore, zebrafish are a useful and popular animal model for a variety of inflammation studies. In in vivo anti-inflammation test models, examining zebrafish is widely accepted as the best method for effective anti-inflammation assay [19,20,21]. Therefore, in this study, extract of soft coral *Dendronephthya puetteri* (DPE) was investigated to identify in vivo anti-inflammatory effects in zebrafish model for its potential use in natural anti-inflammatory agent. The developmental toxicity potential of DPE was also evaluated in a zebrafish model. 

## 2. Results

### 2.1. Effect of DPE on Survival Rate, Heart Beat Rate, and Morphological Changes in Zebrafish Embryo

**Figure 1:**
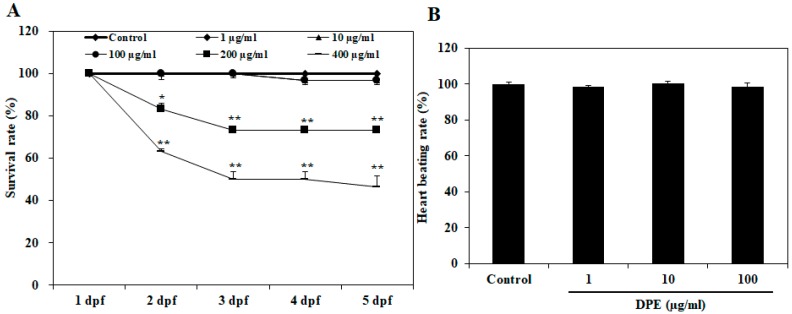
Dose-dependent effect of *Dendronephthya puetteri* (DPE) on zebrafish embryos. (**A**) Survival rates at 1–5 days post-fertilization (dpf) and (**B**) heart beating rates at 2 dpf. The values are expressed as the mean ± SE. Significant differences from the untreated group were identified at * *p* < 0.05 and ** *p* < 0.01.

To determine the toxicity of the DPE, in this study, we observed the survival rate, heart beat rate, and morphological changes in zebrafish embryos following exposure to different concentrations of DPE. As shown in Figure 1A, 1, 10, and 100 µg/mL of DPE did not significantly cause zebrafish embryo death according to assay. Mortality was caused after exposure to 200 and 400 µg/mL of DPE at two days post-fertilization (dpf), respectively (Figure 1A). Notably, 400 µg/mL of DPE caused approximately 40% embryo mortality at 2 dpf. We did not investigate the zebrafish embryos of 200 and 400 µg/mL for further analyses due to the fact that the lethal toxicity was too high. In the heart beat rate test, there was no significant change in heart beat rate compared to control, indicating that there was no toxicity at the tested concentrations (Figure 1B).

**Figure 2:**
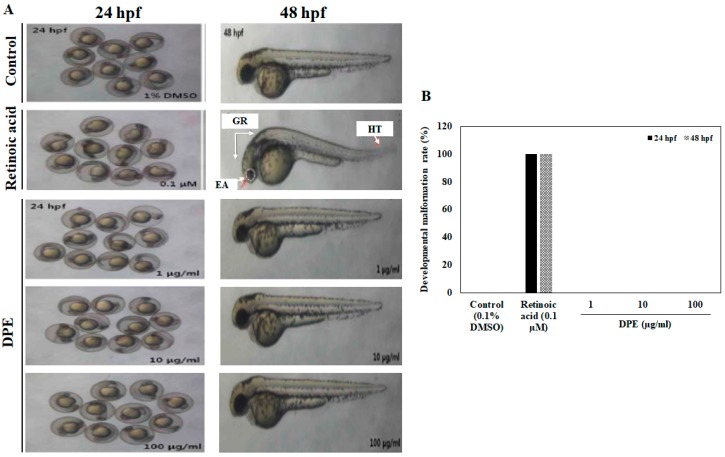
The (**A**) developmental malformations and (**B**) malformation rates in zebrafish embryos exposed to indicated concentrations of DPE at 24 and 48 hpf. GR, general retardation; HT, helical tail; EA, eye alteration. Retinoic acid was employed as a positive control.

To examine the morphologic defects caused by DPE, the developmental abnormalities of zebrafish embryos exposed to DPE were analyzed at 24 and 48 hpf. As shown in Figure 2, no morphological abnormalities in zebrafish embryos were observed at the tested concentrations of DPE, indicating that DPE did not any lead to any toxic effects on the developmental stages of zebrafish embryos. However, upon exposure to 0.1 µM retinoic acid at 24 and 48 hpf, several developmental abnormalities were observed, including general retardation, helical tail, and eye alteration.

### 2.2. Effect of DPE on Cell Death in Zebrafish Embryos

**Figure 3:**
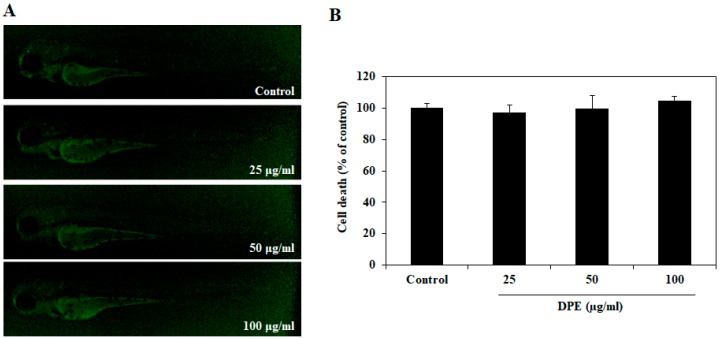
Effect of DPE on cell death in zebrafish embryos. (**A**) The cell death levels were measured after acridine orange staining by image analysis and fluorescence microscopy. (**B**) Individual zebrafish fluorescence intensity was quantified using an image J program. The values are expressed as the mean ± SE.

To evaluate whether DPE has a toxic effect on the cells, zebrafish embryos were treated with DPE for 72 h, and cell death was then measured via acridine orange staining assay. DPE was not exerting any cytotoxic effects at the indicated concentrations (25, 50, and 100 µg/mL) in zebrafish embryos (Figure 3). Based on the results of the preliminary studies, we selected the DPE concentrations as 25, 50, and 100 µg/mL for further experimentation.

### 2.3. In Vivo Effect of DPE on LPS-Induced ROS Generation

**Figure 4:**
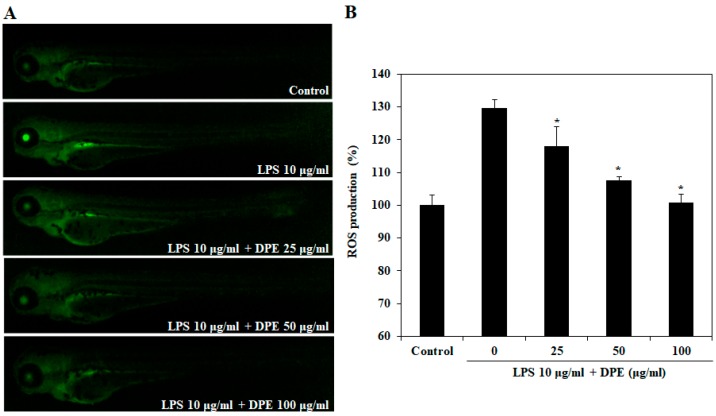
Inhibitory effect of DPE on lipopolysaccharide (LPS)-stimulated ROS production in zebrafish embryos. (**A**) The ROS levels were measured after staining with DCF-DA by image analysis and fluorescence microscopy. (**B**) Individual zebrafish fluorescence intensity was quantified using an image J program. The values are expressed as the mean ± SE. Significant differences from the only LPS-treated group were identified at * *p* < 0.05.

We investigated LPS-induced ROS generation in zebrafish embryos using oxidation sensitive fluorescent probe dye, DCF-DA. Figure 4 shows the protective effect of DPE on LPS-induced ROS generation. The control, which contained no LPS or DPE, generated a clear image, whereas embryos treated only with LPS generated fluorescent images, suggesting that the generation of ROS took place in the presence of LPS in the zebrafish embryos. However, when the zebrafish embryos were treated with DPE prior to LPS treatment, a dose-dependent reduction in the generation of ROS was observed. 

### 2.4. In Vivo Protective Effect of DPE on LPS-Induced Cell Death

**Figure 5:**
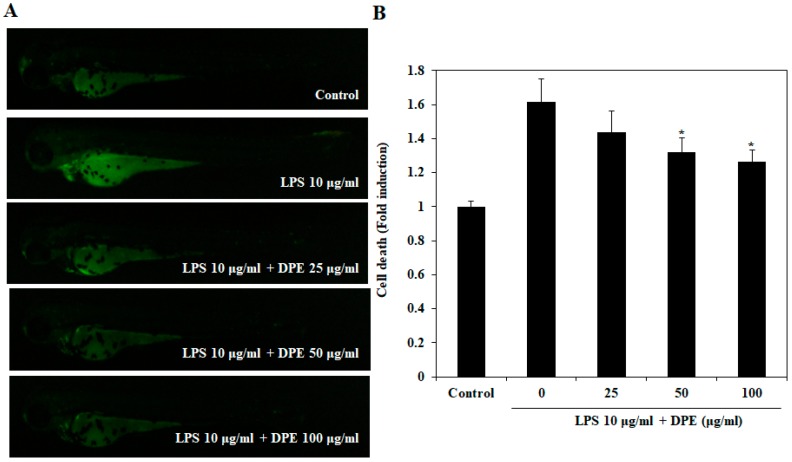
Protective effect of DPE on LPS-stimulated cell death in zebrafish embryos. (**A**) The cell death levels were measured after staining with acridine orange by image analysis and fluorescence microscopy. (**B**) Individual zebrafish fluorescence intensity was quantified using an image J program. The values are expressed as the mean ± SE. Significant differences from the only LPS-treated group were identified at * *p* < 0.05.

The protective effect of DPE on LPS-induced cell death is shown in Figure 5. Cell death in zebrafish embryos was significantly elevated by LPS treatment as compared to cell death in non-LPS treated zebrafish embryos. However, the LPS-induced cell death in DPE-treated zebrafish embryos was significantly reduced in a dose-dependent manner.

### 2.5. In Vivo Effect of DPE on LPS-Induced NO Production

**Figure 6:**
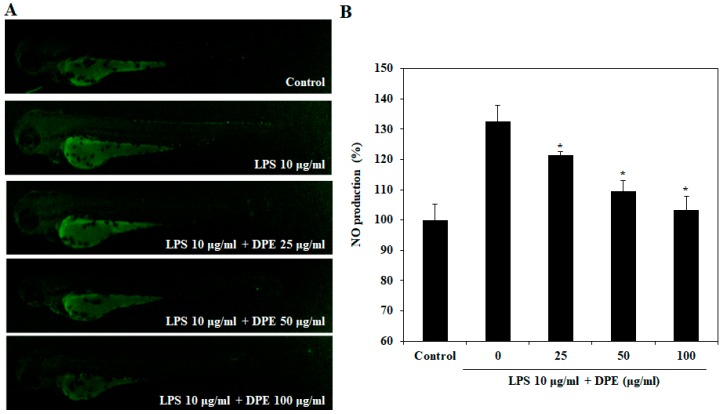
Inhibitory effect of DPE on LPS-stimulated NO production in zebrafish embryos. (**A**) The NO levels were measured after staining with DAF-FM-DA by image analysis and fluorescence microscopy. (**B**) Individual zebrafish fluorescence intensity was quantified using an image J program. The values are expressed as the mean ± SE. Significant differences from the only LPS-treated group were identified at * *p* < 0.05.

The effect of DPE on LPS-induced NO production is shown in Figure 6. Stimulation of the zebrafish embryos with LPS resulted in the enhancement of NO production. However, pretreatment of zebrafish embryos with the DPE significantly decreased the NO production in a dose-dependent manner.

### 2.6. In Vivo Effect of DPE on LPS-Induced Expression of Inflammatory Mediators and Pro-inflammatory Cytokines

**Figure 7:**
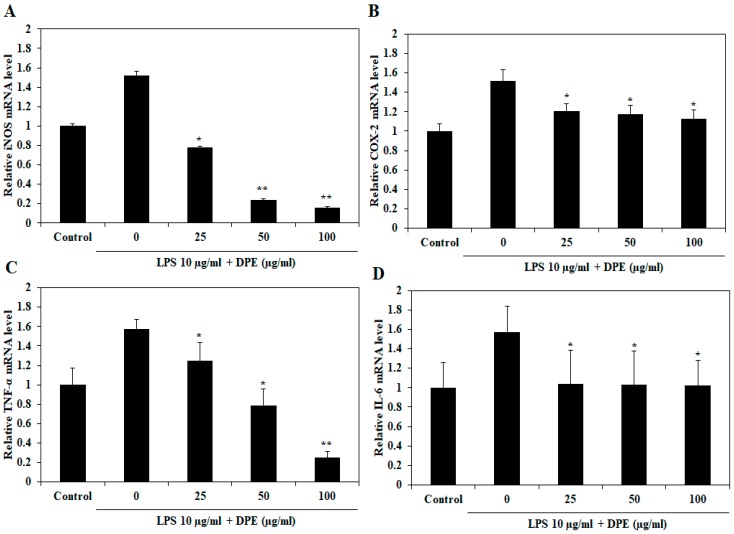
Effect of DPE on expression of iNOS, COX-2, TNF-α, and IL-6 mRNA in LPS-stimulated zebrafish embryos. Zebrafish embryos were stimulated with LPS in the presence of DPE for 72 hpf. The mRNA expression of (**A**) iNOS, (**B**) COX-2, (**C**) TNF-α, and (**D**) IL-6 were evaluated through RT-PCR. The values are expressed as the mean ± SE. Significant differences from the untreated group were identified at * *p* < 0.05 and ** *p* < 0.01.

As shown in Figure 7, treatment with DPE significantly suppressed the mRNA expression of LPS-induced iNOS (Figure 7A) and TNF-α (Figure 7C) in a dose-dependent manner, while COX-2 (Figure 7B) and IL-6 (Figure 7D) mRNA expression significantly reduced relative to the only LPS-treated group treated at all concentrations. Especially, DPE at 100 µg/mL completely suppressed iNOS and TNF-α mRNA expression, whereas the DPE had less effect on the expression of COX-2 and IL-6 mRNA. The downregulation of inflammatory mediators and pro-inflammatory cytokines expression explains the ability of DPE to reduce inflammation in LPS-stimulated zebrafish embryos. 

## 3. Discussion

Several studies have revealed the anti-inflammatory properties of soft corals [22,23,24]. However, to date there has been a lack of information regarding the anti-inflammatory activities and toxicity of soft corals in in vivo models for their potential use as a natural anti-inflammatory agent. Recent studies have reported that zebrafish was used to rapidly and simply assess anti-inflammatory activity against LPS-stimulated inflammation and toxicity [17,20]. Hence, the aim of the present investigation was to evaluate the toxicity and anti-inflammatory effect of DPE in the zebrafish embryo in vivo model.

A significant reduction in the survival rate following exposure to 200 and 400 µg/mL of DPE at 2 dpf was observed in our study. However, 1, 10, and 100 µg/mL of DPE did not significantly cause zebrafish embryo death. Therefore, we did not investigate the zebrafish embryo of 200 and 400 µg/mL for further analyses due to the fact that the lethal toxicity was too high. The heart is one of the first functional organs developed in zebrafish, and the heart beat rate is an important toxicology end point in embryonic testing, so the measurement of heart beat rate is also an important variable of interest in assessing cardiac toxicity [25]. Our results showed that exposure to DPE did not affect the heart beat rate of zebrafish embryo and that there was no significant change compared to the control, indicating that there is no toxicity at the tested concentrations. Based on the toxicological outcomes obtained from our study, we explored the developmental toxicity of DPE through observing the morphologic defects in zebrafish embryos. In toxicity syndromes, morphological alterations usually follow molecular and biochemical changes [26]. Our results showed that no morphological abnormalities in zebrafish embryos were observed at the tested concentrations of DPE. These results clearly show that DPE did not have any toxic effects on the developmental stages of the zebrafish embryos.

A high ROS level induces oxidative stress, which can result in the development of a variety of cell or tissue injuries associated with degenerative diseases, including inflammation. Our results showed that treating zebrafish embryos with LPS-treatment significantly increased ROS levels. However, DPE inhibited this LPS-induced ROS generation. Such cellular damage frequently impairs metabolic function and results in cell death [27]. In the present study, we found that DPE protected against LPS-induced cytotoxic effects in zebrafish embryos. These findings indicate that DPE might confer important protection against the skin inflammation and cellular damage induced by the inhibition of oxidative stress induced by ROS generation. 

NO is an important inflammatory mediator that is synthesized from arginine by nitric oxide synthase (NOS). Under pathological conditions, NO production is increased by the inducible NOS (iNOS), subsequently leading to cytotoxicity and tissue damage [28]. Therefore, NO inhibitors are essential for the prevention of inflammatory diseases. Previous studies have indicated that the extract of soft coral *D. puetteri* collected from Jeju Island suppressed NO generation in murine macrophage cells [11]. In the present study, we found that DPE also significantly reduced the elevated NO level induced by LPS-treatment in zebrafish embryos. The previous and present results indicate that DPE alleviated inflammation by inhibiting NO generation. 

Inflammatory responses are mediated via a complex system of signaling pathways. Inflammatory mediators, including iNOS and COX-2, produce a large amount NO, which is involved in many chronic diseases associated with inflammation [29]. Thus, reducing the levels of iNOS and COX-2 may be an effective strategy for preventing inflammatory disorders. Presently, the overexpression of iNOS and COX-2 mRNA was observed in LPS-stimulated zebrafish embryos and significantly reduced by DPE. Especially, 100 µg/mL of DPE completely suppressed iNOS mRNA expression, while the DPE had less effect on the expression of COΧ-2 mRNA. Therefore, we suggest that the low amount of NO production can be attributed to the strongly inhibited expression of iNOS caused by the presence of DPE. Deregulated production of pro-inflammatory cytokines, such as TNF-α and IL-6, causes many inflammatory diseases [30,31,32]. In addition, previous studies have demonstrated that the expression of iNOS is stimulated by pro-inflammatory cytokines, such as TNF-α [33]. We presently observed that DPE significantly inhibited the expression of pro-inflammatory cytokines TNF-α mRNA in LPS-stimulated zebrafish embryos, but showed less effect on IL-6 mRNA expression, suggesting that the inhibition of the iNOS/NO pathway by DPE is potentially associated with the attenuation of TNF-α and less mediated by reducing IL-6 expression.

In conclusion, the present study demonstrates that DPE has strong anti-inflammatory activity by inhibiting NO and down-regulating the expression of iNOS and pro-inflammatory cytokines TNF-α in an LPS-stimulated inflammatory zebrafish model, and also by protecting against oxidative stress. This outcome can help explain the potential anti-inflammatory activity of DPE, which may be a strong anti-inflammatory agent that could be useful in treating inflammatory diseases. This provides an impetus to carry out further purification and isolation of these bioactive principals to evaluate their specific bioactive functionalities and potential for the development of therapeutic agents.

## 4. Materials and Methods

### 4.1. Preparation of the Soft Coral Dendronephthya puetteri Extract (DPE)

Soft coral *D. puetteri* were collected from the coast of Jeju Island, Korea, and were identified by Jeju Biodiversity Research Institute, Jeju Technopark. Following surface disinfection with 70% ethanol spray to kill the surface attached microorganisms, the samples were washed with tap water to remove any surface attachments and debris. The samples were then lyophilized and ground to form a powder. Each 20 g sample of the soft coral lyophilized powder underwent extraction using 2 L of 70% ethanol at 25 °C for 24 h. Extraction was carried out three times for each sample. Finally, the filtered extracts were concentrated using a rotary evaporator under vacuum.

### 4.2. Origin and Maintenance of Parental Zebrafish

Adult zebrafish were obtained from a commercial dealer (Seoul aquarium, Seoul, Korea) and 10 fishes were kept in a 3 L acrylic tank with the following: At 28.5 °C under a 14:10 h light:dark cycle. The zebrafish were fed three times a day, six days/week, with tetramin flake food supplemented with live brine shrimps (*Artemia salina*; SEWHAPET food Co., Seoul, Korea). Embryos were obtained through natural spawning that was induced in the morning by turning on the light. The collection of embryos into petri dishes was completed within 30 min.

### 4.3. Measurement of Embryo Toxicity

Zebrafish embryos at 7–9 h post-fertilization (hpf) were randomly distributed in 12-well plates at a density of 10 embryos/well, with each well containing 2 mL of embryo medium. The embryos were treated with DPE for 72 h at concentrations of 1, 10, 100, 200, and 400 µg/mL. The final DMSO concentrations was 0.1% in the treatment solution and so 0.1% DMSO was used as a vehicle control during the assays. The medium was not renewed throughout the experiment.

Survival rate was monitored daily throughout the experiment. Any dead embryos were removed every day until 72 hpf. For cardiac toxicity measurement, embryos were anesthetized in 0.4% (*w/v*) tricaine at 48 hpf. The heart-beating rate was measured over 3 min using a microscope (Olympus, Tokyo, Japan), and the results are represented as the average heart-beating rate per min [21]. Cell death was detected in live embryos using acridine orange staining at 72 hpf [34]. For teratotoxicity assay, after zebrafish embryos were exposed to DPE for 24 and 48 hpf, non-lethal malformations were observed under the microscope (Leica, Leica Microsystems, Bannockburn, IL, USA). Retinoic acid was employed as a positive control. All experiments were carried out in triplicate. 

### 4.4. Evaluation of Cell Death and Generation of Intracellular Reactive Oxygen Species (ROS) and Nitric Oxide (NO) in LPS-Stimulated Zebrafish Embryo

Synchronized zebrafish embryos were collected and arrayed by pipette, 15 embryos/well, in 12-well plates containing 2 mL embryo medium for 7–9 hpf, and then incubated with or without DPE for 1 h. To induce inflammation, the embryos were exposed to 10 µg/mL LPS dissolved in the embryo medium for 24 hpf at 28.5 °C. Thereafter, zebrafish embryos were transferred into fresh embryo medium, where they developed for up to 72 hpf. The cell death, intracellular ROS, and NO generation in zebrafish embryos were estimated according to previously reported methods [21,34]. At 72 hpf, the zebrafish embryos were transferred into 24-well plates and separately stained with specific fluorescent probe dyes to determine cell death (acridine orange), intracellular ROS (2’,7’-dichlorodihydrofluorescein diacetate (DCF-DA)), and NO generation (diamino-fluorophore 4-amino-5-methylamino-2’,7’-difluorofluorescein diacetate (DAF-FM DA)). Following incubation for a specified period in the dye-containing media, the embryos were rinsed with fresh embryo media and anesthetized prior to observation, then observed under a fluorescence microscope, which was equipped with a CoolSNAP-Pro color digital camera (Olympus, Tokyo, Japan). The images of the stained embryos were analyzed for cell death, intracellular ROS, and NO generation, and the fluorescence intensities of individual embryos were quantified using ImageJ 1.46r software (Wayne Rasband, National Institutes of Health, Bethesda, MD, USA). Cell death, intracellular ROS, and NO generation were calculated by comparing the fluorescence intensities of the treatment embryos to the controls.

### 4.5. Quantitative Real-Time Polymerase Chain Reaction (PCR) Analysis

Total RNA was extracted from zebrafish embryos using the RNeasy mini kit (Qiagen, Hilden, Germany). For real-time PCR, first-strand complementary DNA (cDNA) was synthesized from 1 μg total RNA using the Advantage RT-for-PCR Kit (Clontech, Palo Alto, CA, USA). Relative messenger RNA levels were determined by real-time PCR using LightCycler 480 SYBR Green I Mater mix and a LightCycler 480 (Roche, Mannheim, Germany). All cDNA levels were normalized to the level of ubiquitin cDNA. Samples were amplified using the following sense primer and antisense primer: Forward 5′-TAGAACAACCCAGCAAAC-3′ and reverse 5′-ACCAGCGGTAAAGGCAAC-3′ for TNF-α; forward 5′-AGCCCTACTCATCCTTTGAGG-3′ and reverse 5′-TCAACCTTGTCTACGTGACCATA-3′ for COX-2; forward 5′-GCCAAC AGAGAGAAGATGAC-3′ and reverse 5′-GGAAAAGCTCAGTGACTT-3′ for iNOS; forward 5′-GAGGATACCACTCCCAACAG-3′ and reverse 5′-AAGTGCATCATCGTTGTTCATACA-3′ for IL-6; forward 5′-GCCAACAGAGAGAAGATGAC-3′ and reverse 5′-CACCAGAGTCCATCACAATAC-3′ for β-actin. 

### 4.6. Statistical Analysis

The data are presented as means ± standard error (SE). Statistical comparisons of the mean values were performed by analysis of variance (ANOVA), followed by a Duncan’s multiple range test using SPSS software. Statistical significance was considered at *p* < 0.05.

## References

[1] Palladino M.A., Bahjat F.R., Theodorakis E.A., Moldawer L.L. (2003). Anti-TNF-alpha therapies: The next generation. Nat. Rev. Drug Discov..

[2] Dinarello C.A. (1997). Proinflammatory and anti-inflammatory cytokines as mediators in the pathogenesis of septic shock. Chest.

[3] Serhan C.N., Savill J. (2005). Resolution of inflammation: the beginning programs the end. Nat. Immunol..

[4] Oikarinen A., Haapasaari K.M., Sutinen M., Tasanen K. (1998). The molecular basis of glucocorticoid-induced skin atrophy: Topical glucocorticoid apparently decreases both collagen synthesis and the corresponding collagen mRNA level in human skin in vivo. Br. J. Dermatol..

[5] Lakshmi V., Kumar R. (2009). Metabolites from Sinularia species. Nat. Prod. Res..

[6] Blunt J.W., Copp B.R., Keyzers R.A., Munroa M.H.G., Prinsep M.R. (2012). Marine natural products. Nat. Prod. Rep..

[7] Elkhayat E.S., Ibrahim S.R.M., Fouad M.A., Mohamed G.A. (2014). Dendronephthols A-C, new sesquiterpenoids from the Red Sea soft coral *Dendronephthya* sp.. Tetrahedron.

[8] Chao C.H., Wen Z.H., Chen I.M., Su J.H., Huang H.C., Chiang M.Y., Sheu J.H. (2008). Anti-inflammatory steroids from the octocoral *Dendronephthya griffin*. Tetrahedron.

[9] Li G., Deng Z., Guan H., van Ofwegen L., Proksch P., Lin W. (2005). Steroids from the soft coral Dendronephthya sp.. Steroids.

[10] Tomono Y., Hirota H., Imahara Y., Fusetani N.J. (1999). Four new steroids from two octocorals. J. Nat. Prod..

[11] Wang L., Oh J.Y., Shanura Fernando I.P., Asanka Sanjeewa K.K., Kim E.A., Lee W.W., Jeon Y.J. (2016). Soft corals collected from Jeju Island; a potential source of anti-inflammatory phytochemicals. J. Chitin Chitosan.

[12] Shanura Fernando I.P., Asanka Sanjeewa K.K., Kim H.S., Kim S.Y., Lee S.H., Lee W.W., Jeon Y.J. (2017). Identification of sterols from the soft coral *Dendronephthya gigantea* and their anti-inflammatory potential. Environ. Toxicol. Pharmacol..

[13] Shanura Fernando I.P., Asanka Sanjeewa K.K., Kim H.S., Wang L., Lee W.W., Jeon Y.J. (2018). Apoptotic and antiproliferative properties of 3β-hydroxy-Δ5-steroidal congeners from a partially purified column fraction of *Dendronephthya gigantean* against HL-60 and MCF-7 cancer cells. J. Appl. Toxicol..

[14] Eisen J.S. (1996). Zebrafish make a big splash. Cell.

[15] Fishman M.C. (1999). Zebrafish genetics: The enigma of arrival. Proc. Natl. Acad. Sci. USA.

[16] Ali S., Champagne D.L., Spaink H.P., Richardson M.K. (2011). Zebrafish embryos and larvae: A new generation of disease models and drug screens. Birth Defects Res. C Embryo Today.

[17] He J.H., Guo S.Y., Zhu F., Zhu J.J., Chen Y.X., Huang C.J., Gao J.M., Dong Q.X., Xuan Y.X., Li C.Q. (2013). A zebrafish phenotypic assay for assessing drug-induced hepatotoxicity. J. Pharmacol. Toxicol. Methods.

[18] den Hertog J. (2005). Chemical genetics: Drug screens in zebrafish. Biosci. Rep..

[19] Liao Y.F., Chiou M.C., Tsai J.N., Wen C.C., Wang Y.H., Cheng C.C., Chen Y.H. (2011). Resveratrol treatment attenuates the wound-induced inflammation in zebrafish larvae through the suppression of myeloperoxidase expression. J. Food Drug Anal..

[20] Park K.H., Cho K.H. (2011). A zebrafish model for the rapid evaluation of pro-oxidative and inflammatory death by lipopolysaccharide, oxidized low-density lipoproteins, and glycated high-density lipoproteins. Fish Shellfish Immunol..

[21] Lee S.H., Ko C.I., Jee Y., Jeong Y., Kim M., Kim J.S., Jeon Y.J. (2013). Anti-inflammatory effect of fucoidan extracted from *Ecklonia cava* in zebrafish model. Carbohydr. Polym..

[22] Fenical W. (1987). Marine soft corals of the genus Pseudopterogorgia: A resource for novel anti-inflammatory diterpenoids. J. Nat. Prod..

[23] Hu J., Yang B., Lin X., Zhou X., Yang X., Long L., Liu Y. (2011). Chemical and biological studies of soft corals of the Nephtheidae family. Chem. Biodivers..

[24] Radhika P., Rao P.R., Archana J., Rao N.K. (2005). Anti-inflammatory activity of a new sphingosine derivative and cembrenoid diterpene (lobohedleolide) isolated from marine soft corals of *Sinularia crassa* Tixier-Durivault and *Lobophytum* species of the Andaman and Nicobar Islands. Biol. Pharm. Bull..

[25] De L.E., Zaccaria G.M., Hadhoud M., Rizzo G., Ponzini R., Morbiducci U., Santoro M.M. (2014). ZebraBeat: A flexible platform for the analysis of the cardiac rate in zebrafish embryos. Sci. Rep..

[26] Liu H., Gooneratne R., Huang X., Lai R., Wei J., Wang W. (2015). A rapid in vivo zebrafish model to elucidate oxidative stress-mediated PCB126-induced apoptosis and developmental toxicity. Free Radic. Biol. Med..

[27] Finkel T.N., Holbrook J. (2000). Oxidants, oxidative stress and the biology of aging. Nature.

[28] Kim H.K., Cheon B.S., Kim Y.H., Kim S.Y., Kim H.P. (1999). Effects of naturally occurring flavonoids on nitric oxide production in the macrophage cell line RAW 264.7 and their structure-activity relationships. Biochem. Pharmacol..

[29] Lee S.H., Ko C.I., Ahn G., You S.G., Kim J.S., Heu M.S., Kim J., Jee Y., Jeon Y.J. (2012). Molecular characteristics and anti-inflammatory activity of the fucoidan extracted from *Ecklonia cava*. Carbohydr. Polym..

[30] Dinarello C.A. (2000). Proinflammatory cytokines. Chest.

[31] De Nardin E. (2001). The role of inflammatory and immunological mediators in periodontitis and cardiovascular disease. Ann. Periodontol..

[32] Hirano T. (1998). Interleukin 6 and its receptor: Ten years later. Int. Rev. Immunol..

[33] Marcus J.S., Karackattu S.L., Fleegal M.A., Sumners C. (2003). Cytokine-stimulated inducible nitric oxide synthase expression in astroglia: Role of Erk mitogen-activated protein kinase and NF-κB. Glia.

[34] Kang M.C., Cha S.H., Wijesinghe W.A.J.P., Kang S.M., Lee S.H., Kim E.A., Song C.B., Jeon Y.J. (2013). Protective effect of marine algae phlorotannins against AAPH-induced oxidative stress in zebrafish embryo. Food Chem..

